# La rupture spontanée de carcinome hépatocellulaire: un diagnostic d'urgence à connaître

**DOI:** 10.11604/pamj.2015.22.65.7937

**Published:** 2015-09-23

**Authors:** Ammar Mahmoudi, Mezri Maâtouk

**Affiliations:** 1 Service de chirurgie générale et digestive, CHU Fattouma Bourguiba de Monastir, Tunisie; 2 Service d'imagerie médicale, CHU Fattouma Bourguiba de Monastir, Tunisie

**Keywords:** Foie, carcinome hépatocellulaire, scanner, embolisation, chimioembolisation intra-artérielle hépatique, liver, hepatocellular carcinoma:, CT scan, embolization, intra-arterial hepatic chemoembolization

## Image en medicine

La rupture spontanée de carcinome hépatocellulaire (CHC) représente une complication relativement rare et potentiellement fatale. Les situations cliniques sont différentes depuis l'hémorragie compensée jusqu’à la rupture péritonéale massive entraînant un hémopéritoine abondant mettant en jeu le pronostic vital. Le diagnostic repose sur le scanner qui détecte la tumeur et l’éventuelle extravasation si le saignement est actif. La mise en évidence de l'extravasation se fait parfois au temps artériel, mais elle est presque toujours visible au temps portal. L'embolisation est le premier traitement en urgence à envisager dans la rupture de CHC ce qui ne préjuge pas d'une autre prise en charge ultérieure en cas d’évolution favorable. Avec un taux de succès dépassant 90%, il s'agit d'une méthode peu invasive en comparaison avec la chirurgie. On peut effectuer soit une embolisation simple, soit d'emblée une chimio-embolisation lipiodolée. Nous rapportons l'observation d'un patient âgé de 60 ans chez qui a été découvert il y a un mois un CHC de 7 cm du foie gauche sur foie cirrhotique. Il avait présenté cinq heures avant sa consultation une épigastralgie brutale et intense associée à une distension abdominale, un retentissement hémodynamique et une déglobulisation (Hb:7,5 g/dl). Le scanner abdominal (A, B) a montré un CHC du foie gauche compliqué d'hémorragie intrapéritonéale de moyenne abondance avec une extravasation du produit de contraste en flaque péritumorale. Après transfusion sanguine et stabilisation de son état hémodynamique, il lui a été réalisé une chimio-embolisation (C, D) permettant de contrôler le saignement. Les suites immédiates étaient favorables.

**Figure 1:**
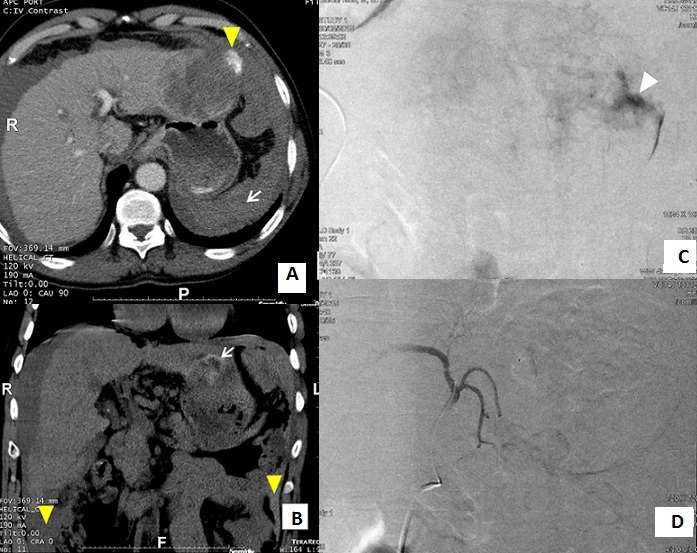
(A) TDM abdominale après injection de produit de contraste au temps portal: masse tissulaire protrusive du lobe gauche hypodense hétérogène associée à un épanchement intra-péritonéale avec composante hyperdense en rapport avec un hémopéritoine (flèche) avec fuite active du produit de contraste en regard d'une perte de contour focale de la masse (tête de flèche); (B) TDM abdominale en contraste spontané (reconstruction coronale): masse sous capsulaire du lobe gauche de densité hétérogène avec des plages hyperdenses en son sein en rapport avec un saignement récent (flèche) associée à un hémopéritoine (tête de flèche); (C) artériographie hépatique pré-embolisation: blush tumoral (correspondant au carcinome hépatocellulaire) avec saignement actif (tête de flèche); (D) artériographie hépatique (branche gauche) post-embolisation: absence de saignement actif

